# Alterations in Kainate Receptor and TRPM1 Localization in Bipolar Cells after Retinal Photoreceptor Degeneration

**DOI:** 10.3389/fncel.2015.00486

**Published:** 2015-12-22

**Authors:** Jacqueline Gayet-Primo, Theresa Puthussery

**Affiliations:** Casey Eye Institute, Department of Ophthalmology, Oregon Health and Science University, PortlandOR, USA

**Keywords:** *rd10*, human retina, mouse retina, Neto1, GluK1

## Abstract

Photoreceptor degeneration differentially impacts glutamatergic signaling in downstream On and Off bipolar cells. In rodent models, photoreceptor degeneration leads to loss of glutamatergic signaling in On bipolar cells, whereas Off bipolar cells appear to retain glutamate sensitivity, even after extensive photoreceptor loss. The localization and identity of the receptors that mediate these residual glutamate responses in Off bipolar cells have not been determined. Recent studies show that macaque and mouse Off bipolar cells receive glutamatergic input primarily through kainate-type glutamate receptors. Here, we studied the impact of photoreceptor degeneration on glutamate receptor and their associated proteins in Off and On bipolar cells. We show that the kainate receptor subunit, GluK1, persists in remodeled Off bipolar cell dendrites of the rd10 mouse retina. However, the pattern of expression is altered and the intensity of staining is reduced compared to wild-type retina. The kainate receptor auxiliary subunit, Neto1, also remains in Off bipolar cell dendrites after extensive photoreceptor degeneration. Similar preservation of kainate receptor subunits was evident in human retina in which photoreceptors had degenerated due to serous retinal detachment. In contrast, photoreceptor degeneration leads to loss of synaptic expression of TRPM1 in mouse and human On bipolar cells, but strong somatic expression remains. These findings demonstrate that Off bipolar cells retain dendritic glutamate receptors during retinal degeneration and could thus serve as a conduit for signal transmission from transplanted or optogenetically restored photoreceptors.

## Introduction

Retinal diseases such as retinitis pigmentosa and age-related macular degeneration culminate in the loss of rod and cone photoreceptors, leading to visual impairment. Photoreceptor degeneration also leads to downstream morphological and functional changes in the inner retina. For instance, de-afferentation leads to changes in the second-order bipolar cells including: dendritic remodeling, loss and mislocalization of glutamate receptors and formation of ectopic synaptic contacts (Gargini et al., 2007; Barhoum et al., 2008; Puthussery et al., 2009). These changes could impede strategies aimed at vision restoration. For example, efforts to replace photoreceptors or restore their function may be inadequate if bipolar cells cannot maintain functional glutamatergic synapses (Busskamp et al., 2010; Pearson et al., 2012). Conversely, restoration of light sensitivity to bipolar cells through optogenetic or chemical photoswitches (Lagali et al., 2008; Gaub et al., 2014), might be more effective if bipolar cells have lost glutamate responsiveness, thereby reducing the possibility of conflicting signals from dysfunctional photoreceptors. Understanding the localization and functional status of bipolar cell glutamate receptors is thus important for developing treatments for retinal degeneration.

Photoreceptors signal changes in light intensity by altering the rate of glutamate release from their synaptic terminals. On and Off bipolar cells respond to these glutamate fluctuations with opposite polarity due to differences in their glutamate receptors. In On bipolar cells, activation of the metabotropic glutamate receptor 6 (mGluR6) leads to closure of the non-selective cation channel, TRPM1, and membrane hyperpolarization (Nomura et al., 1994; Masu et al., 1995; Morgans et al., 2009; Koike et al., 2010). On the other hand, glutamate depolarizes Off bipolar cells through activation of ionotropic glutamate receptors (Slaughter and Miller, 1983). In mouse models of degeneration, de-afferentation leads to down-regulation of mGluR6 and concomitant loss of On bipolar cell function [(Gargini et al., 2007; Puthussery et al., 2009), but see (Barhoum et al., 2008)]. In contrast, glutamate receptor function in Off bipolar cells appears to be comparatively resistant to degeneration. In the *rd10* mouse, glutamatergic agonists produced robust inward currents in Off bipolar cells even after cone loss (Puthussery et al., 2009). In accord with this finding, Off ganglion cells retain light-evoked signaling longer than On ganglion cells (Pu et al., 2006; Stasheff, 2008; Fransen et al., 2015). These findings suggest that Off bipolar cells might represent a better conduit for signals from transplanted or functionally restored photoreceptors.

What types of receptors mediate glutamatergic currents in Off bipolar cells and where are they located? Although the AMPA receptor subunits GluA1, GluA2, and GluA4 persist in the *rd10* outer retina after photoreceptor degeneration (Puthussery et al., 2009), recent studies indicate that kainate receptors primarily mediate Off bipolar responses in mice and macaques (Puller et al., 2013; Borghuis et al., 2014; Puthussery et al., 2014). Thus, our goal was to evaluate the expression and localization of kainate receptors and their associated auxiliary proteins in Off bipolar cells during the progression of photoreceptor degeneration. A further aim was to determine the impact of photoreceptor degeneration on the localization of TRPM1 in On bipolar cells.

## Materials and Methods

### Animal and Tissue Preparation

Animal use protocols were approved by the OHSU Institutional Animal Care and Use Committee. Wild-type mice were C57BL/6J (JAX stock no 000664), or B6EiC3Sn.BLiAF1/J (JAX stock no *003647*). The latter strain harbors the wild-type allele of *PDE6b* on a mixed background. No differences were noted between these two strains. Homozygote B6.CXB1-*Pde6b^rd10^* mice (JAX stock # 004297, referred to herein as *rd10*) harbor a spontaneous missense mutation in cGMP phosphophodiesterase 6B. Human retinal specimens were obtained from surgical procedures in which eyes were enucleated for management of orbital or retinal pathology. Normal retinal samples were from adult males aged 51 and 72 years-old. One sample was from superior-nasal retina at an eccentricity of 12–15 mm, the other from infero-temporal retina at an eccentricity of approximately 6–9 mm. The degenerated retina was obtained from the far peripheral retina of an adult male in which the eye was enucleated for management of a choroidal melanoma (eccentricity and quadrant not known). In the region obtained, the photoreceptor layer had degenerated due to serous retinal detachment secondary to the melanoma. All human tissue samples were de-identified prior to receipt by the investigators and were thus deemed to be non-human subject research by the OHSU Institutional Review Board.

We studied five *rd10* retinae and four wild-type retinae in the age range of 3–4 months (Cohort 1). At this time-point, some residual photoreceptors were present in the peripheral retina. To assess longer-term changes in receptor expression we also examined staining in an older cohort of animals aged between 8 and 18 months (four *rd10* and six wild-type animals). Despite the wide age range studied, *rd10* samples from within this time point were devoid of residual photoreceptors in the central retina (as assessed with recoverin and vGluT1 staining) and thus were pooled for analysis. No gross age-related protein expression changes were observed in wild-type mice. Immunohistochemistry was performed on sections taken from regions close to the optic nerve.

Mice were anaesthetized with an intraperitoneal injection of sodium pentobarbital and euthanized by cervical dislocation. Eyes were enucleated and placed in fixative containing 2 or 4% paraformaldehyde in 0.1 M phosphate buffer (pH = 7.4). The anterior eyecup and lens were removed and posterior eyecups were fixed for 5 min in 2 or 4% paraformaldehyde or for 30 min in 4% paraformaldehyde. After fixation, eyecups were cryoprotected in graded sucrose solutions (10%, 20%, 30%), embedded in OCT mounting medium (Tissuetek) and frozen rapidly in isopentane cooled to near freezing point in liquid nitrogen. Vertical cryosections were prepared at 14 μm thickness and mounted on Superfrost Plus Slides. Slides were frozen at -20°C until use.

### Antibodies

The following primary antibodies were used at the specified dilutions; goat anti-GluK1 antibody (GluR5, Santa Cruz Biotechnology, SC-7616, 1:100), mouse anti-Islet-1 (Developmental Hybridoma Studies Bank, University of Iowa, #39.4D5, 1:250–500), rabbit anti-neuropilin and tolloid-like 1 (Neto1, kindly provided by Dr M. Watanabe, Hokkaido University, 1:320), rabbit anti-recoverin (Rec1, kindly provided by Dr. G. Adamus, Casey Eye Institute, OR, 1:200), rabbit or sheep anti-secretagogin (Biovendor R&D, #RD181120100, RD184120100, 1:2000–5000), rabbit anti-TRPM1 (Sigma, HPA014785, 1:4500), sheep anti-TRPM1 (kind gift from Dr K. Martemyanov, The Scripps Research Institute, FL, 1:500). Peanut Agglutinin Lectin (Alexa Fluor 488 conjugated) was obtained from Invitrogen/Life Technologies (L21409, 1:800).

### Immunohistochemistry

For immunohistochemistry, retinal sections were blocked for 1 h in a buffer containing 10% normal horse serum, 1% Tx100, 0.025% NaN_3_ in PBS. Primary antibodies were diluted in a buffer containing 3% normal horse serum, 1% Tx100, 0.025% NaN_3_ in PBS and applied to retinal sections in a humidified chamber overnight at 25°C. Sections were washed and then incubated with secondary antibodies diluted in 3% normal horse serum, 0.025% Na_N3_ in PBS for 1 h at 25°C. Anti-mouse, anti-rabbit, anti-goat, and anti-sheep secondary antibodies were raised in donkey and conjugated to Alexa Fluor 488 or Alexa Fluor 594 (Life Technologies, Carlsbad, CA, USA).

### Imaging and Analysis

**FIGURE 1:**
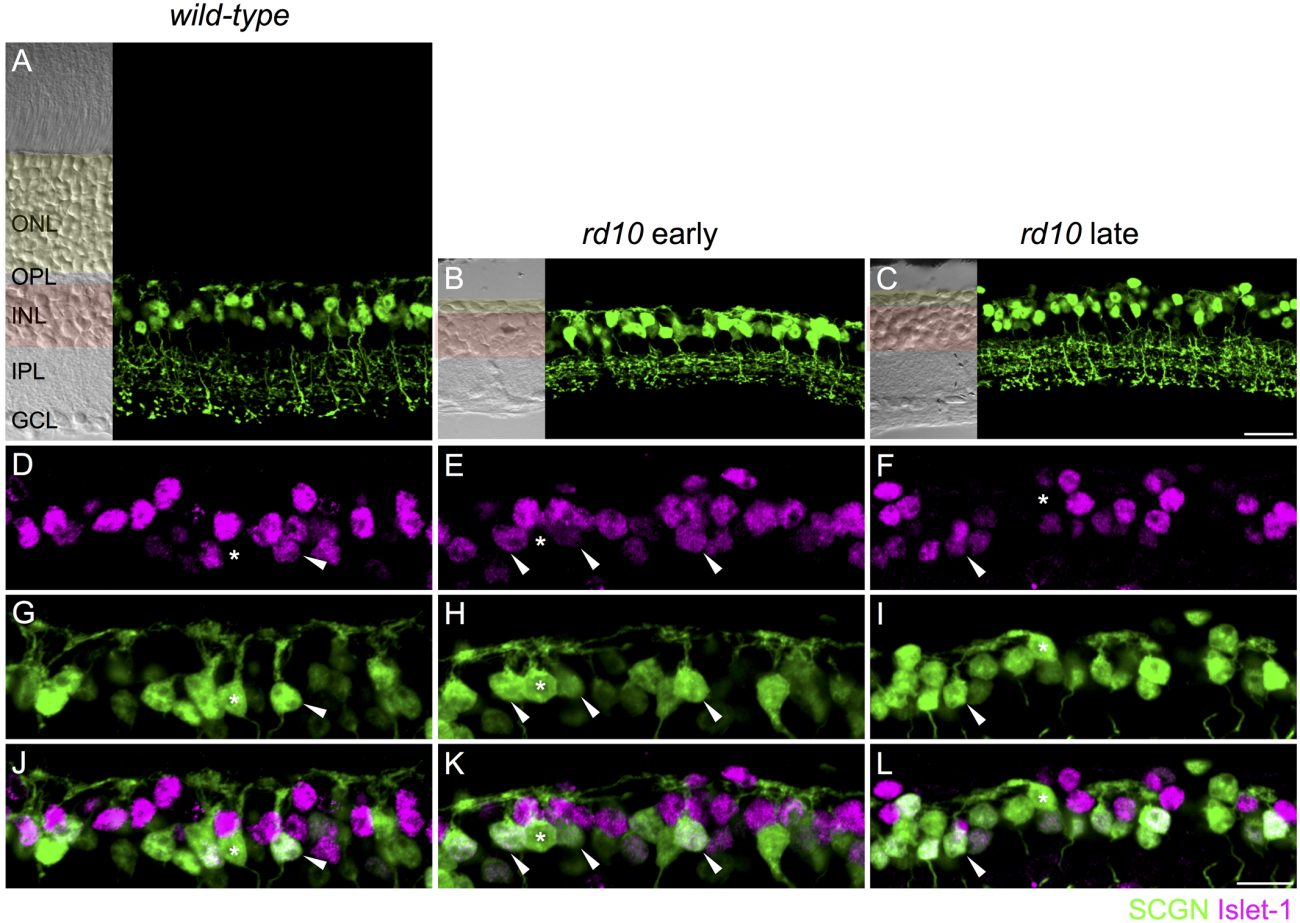
**On and Off cone bipolar cells retain dendrites after extensive photoreceptor degeneration.** **(A–C)** Confocal micrographs showing secretagogin immunoreactive cone bipolar cells in wild-type (**A**, 8 months of age) and *rd10* retina **(B,C)**. Transmitted light images are displayed to the left of each panel to show retinal morphology. The outer nuclear layer (ONL) is shaded in yellow and the inner nuclear layer (INL) in red. OPL, outer plexiform layer; IPL, inner plexiform layer; GCL, ganglion cell layer. **(D–L)** High power micrographs of the outer retina showing double labeling for Islet-1 (**D–F,** magenta) and secretagogin (SCGN, **G–I**, green). Merged images are shown in panels **(J–L)**. Examples of Off cone bipolar cell somata (Islet-1 negative, SCGN positive) with connected dendrites are indicated with asterisks. Examples of On cone bipolar cell somata (Islet-1 positive, SCGN positive) with connected dendrites are indicated with arrowheads. Scale bar in **(C)** (applies to **A–C**) = 20 μm, in **(L)** (applies to **D–L**) = 10 μm.

**FIGURE 2:**
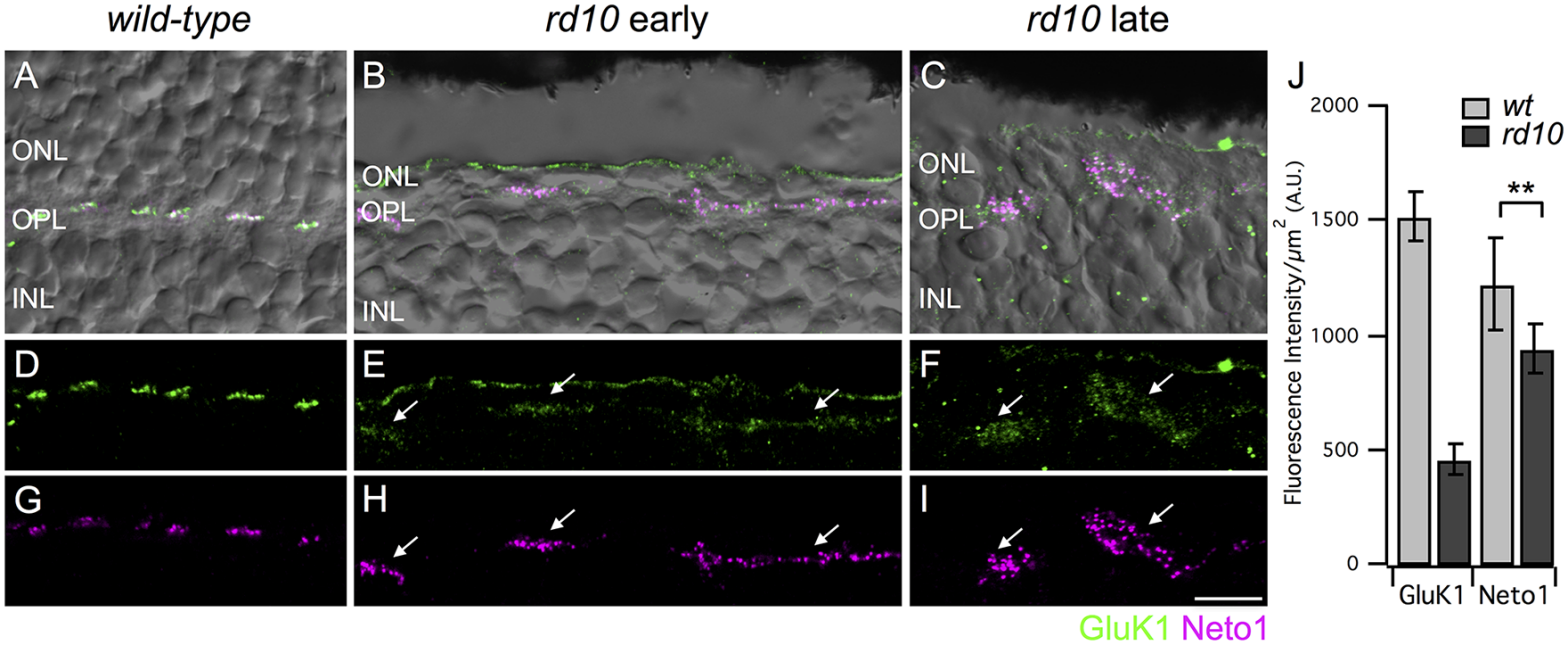
**Persistence of kainate receptors after photoreceptor degeneration.** **(A–C)** Micrographs showing the localization of GluK1 (green) and Neto1 (magenta) overlayed on transmitted light images (gray) of wild-type and *rd10* retina. Clusters of GluK1 and Neto1 were localized to the OPL. Note that some green fluorescence was also evident at the outer edge of the ONL but this staining was not included in the analysis as it was not associated with bipolar cell dendrites. **(D–F)** Same regions as in **(A–C)** showing GluK1 immunoreactivity (arrows) in wild-type and *rd10* retina. Note that for illustration purposes, confocal gain and laser power were increased to show residual GluK1 staining in the *rd10* retina. **(G–I)** Same regions as in **(A–C)** showing clusters of Neto1 immunoreactivity (arrows). Confocal acquisition settings were identical for panels **(G–I)**. **(J)** Quantification of fluorescence intensity for regions-of-interest (ROI) from retinae from the late time point. Each ROI was a polygonal shape surrounding a separate cluster of GluK1/Neto1 puncta. There was a significant reduction in GluK1 immunoreactivity in *rd10* retina compared to wild-type (unpaired *t*-test, ^∗∗^*P* = 0.001). Neto1 intensity was comparable between genotypes (unpaired *t*-test, ^∗∗^*P* = 0.290). Data were obtained from 57 clusters from 3 *rd10* retinae and 176 clusters from 3 wild-type retinae). Scale bar = 10 μm.

**FIGURE 3:**
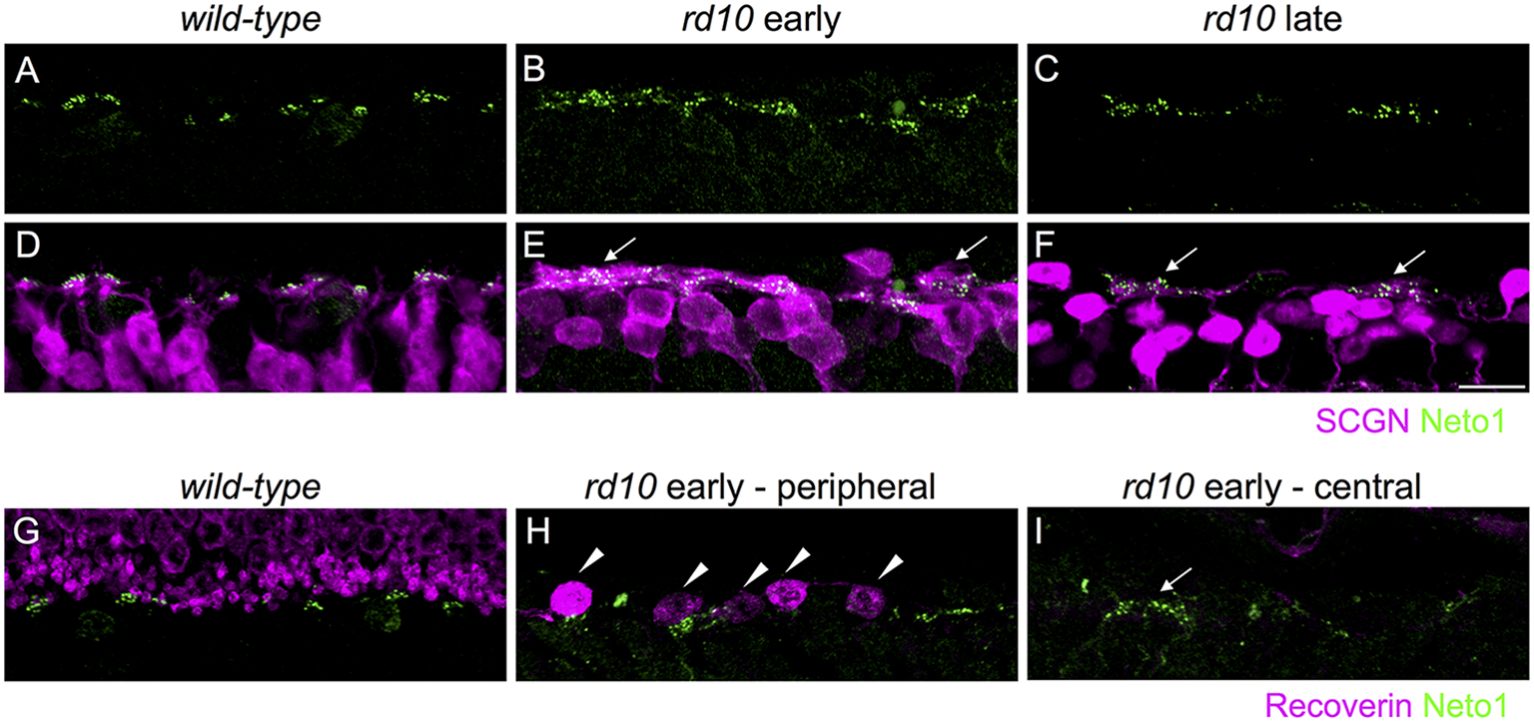
**Kainate receptors cluster in remodeled bipolar cell dendrites and persist in the absence of residual photoreceptors in *rd10* retina.** **(A–C)** Clusters of Neto1 puncta in wild-type and *rd10* outer retina. **(D–F)** Same regions as in **(A–C)** showing double labeling for Neto1 and secretagogin. Note that Neto1 puncta are associated with remodeled cone bipolar cell dendrites in both the early and late *rd10* age groups (arrows). **(G–I)** Regions of outer retina double labeled for recoverin and Neto1 in wild-type and *rd10* retina at the early time-point. Note the recoverin-labeled photoreceptor terminals in the wild-type retina **(G)**. Some residual dysmoprhic cones (arrowheads) remain in the peripheral *rd10* retina **(H)**, however, in the central retina Neto1 clusters (arrow) remain despite the lack of any residual cones. Scale bar = 10 μm.

**FIGURE 4:**
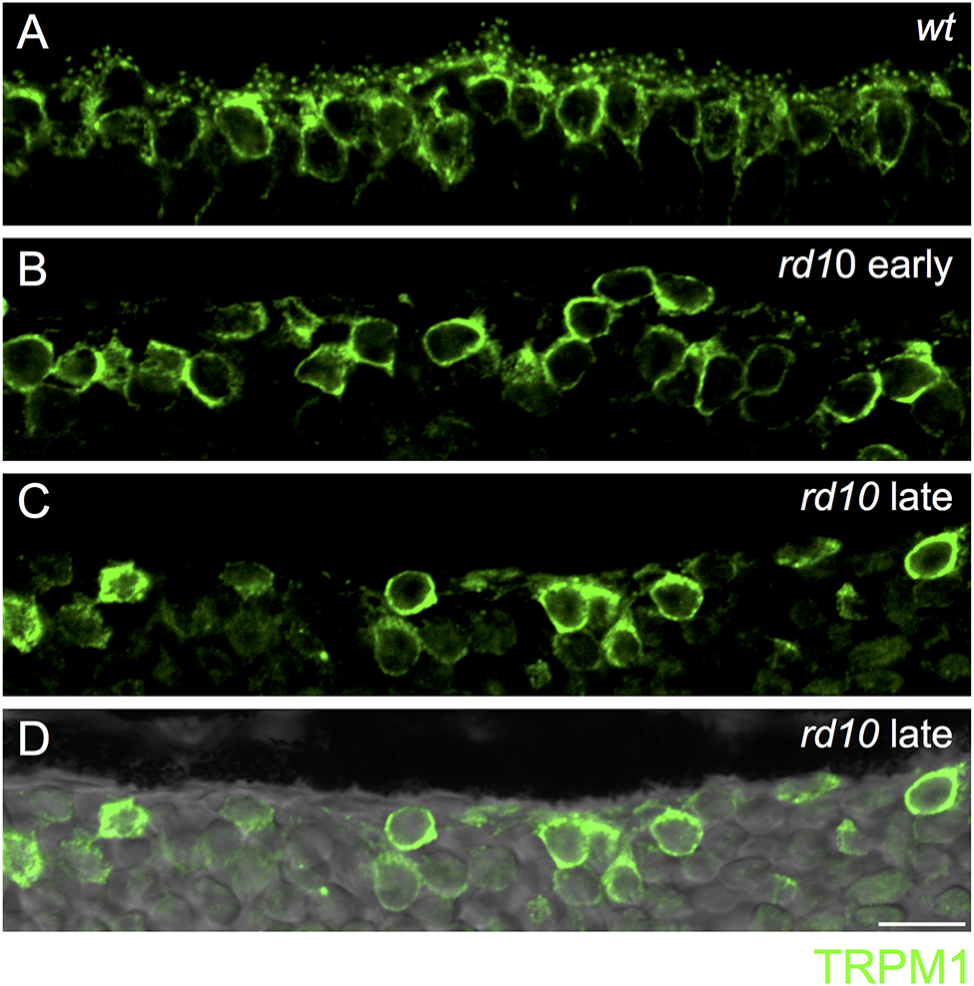
**Loss of synaptic TRPM1 expression in *rd10* retina.** **(A)** Region of outer wild-type retina showing TRPM1 immunoreactivity. Note the strong punctate labeling in the dendritic tips of the bipolar cells. Strong somatic labeling is also evident. **(B–D)** Loss of synaptic TRPM1 expression in the early **(B)** and late **(C)** *rd10* retina. Punctate staining is absent in the rd10 retina indicating a lack of synaptic expression. However, strong labeling remains in On bipolar cell somata. **(D)** Same area as in **(C)** overlayed on the transmitted light image to show the lack of retinal photoreceptors in this region. Scale bar = 10 μm.

**FIGURE 5:**
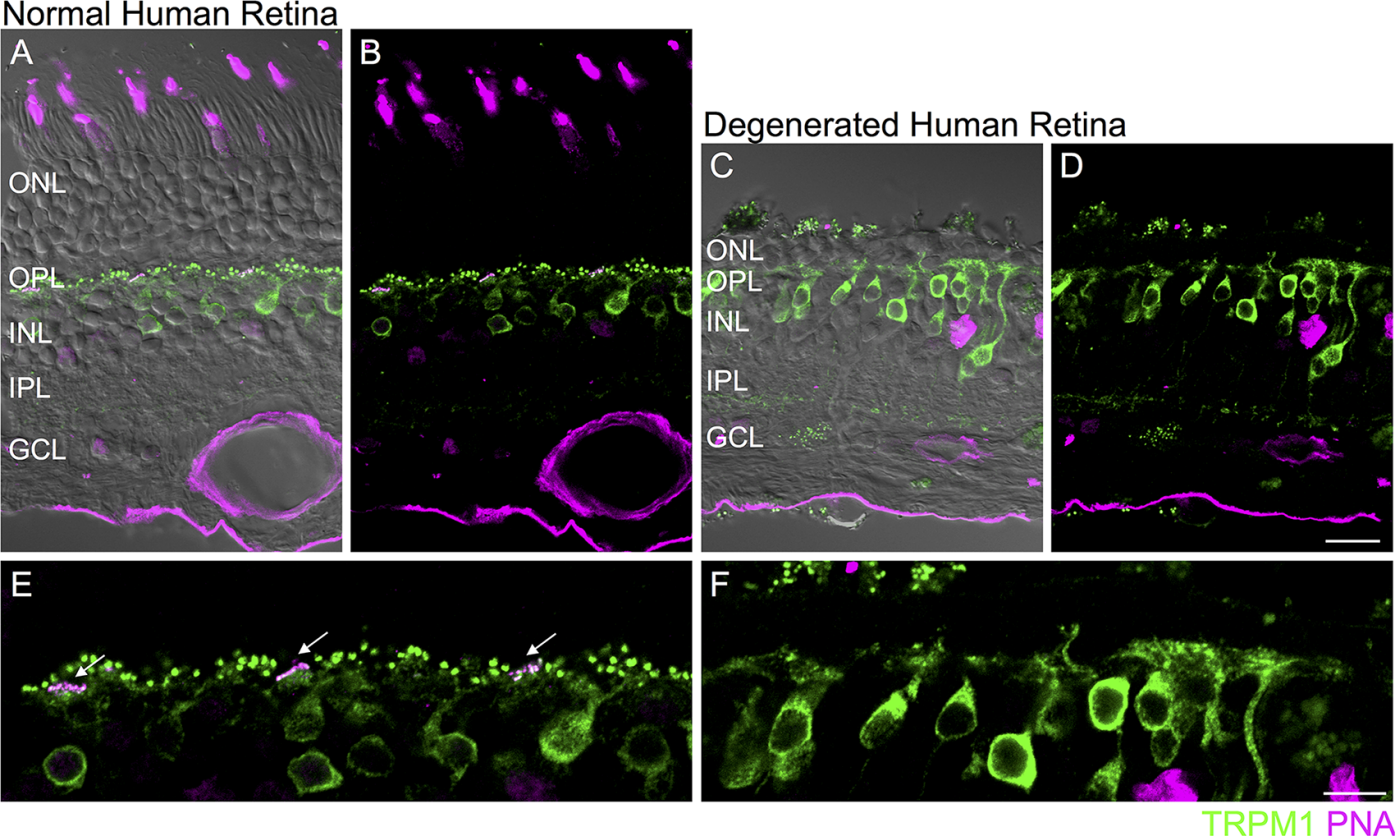
**Loss of synaptic TRPM1 after photoreceptor degeneration in human retina.** **(A–D)** Immunolocalization of TRPM1 (green) and PNA (magenta) in normal human peripheral retina **(A,B)** and in degenerated human retina **(C,D)**. The fluorescence image is superimposed on the transmitted light image (gray) to show retinal morphology. Scale bar = 20 μm. **(E,F)**: High magnification view of the normal **(E)** and degenerated **(F)** outer retina. In the normal retina **(E)**, punctate TRPM1 immunolabeling is present in the outer plexiform layer as well as in the somata and dendritic tips of ON bipolar cells. Clusters of TRPM1 staining are associated with PNA-labeled cone pedicles (arrows). **(F)** Immunolocalization of TRPM1 (green) and PNA (magenta) in human retina after extensive photoreceptor degeneration. There is loss of synaptic punctate staining but strong somatic staining remains. Scale bar = 10 μm.

**FIGURE 6:**
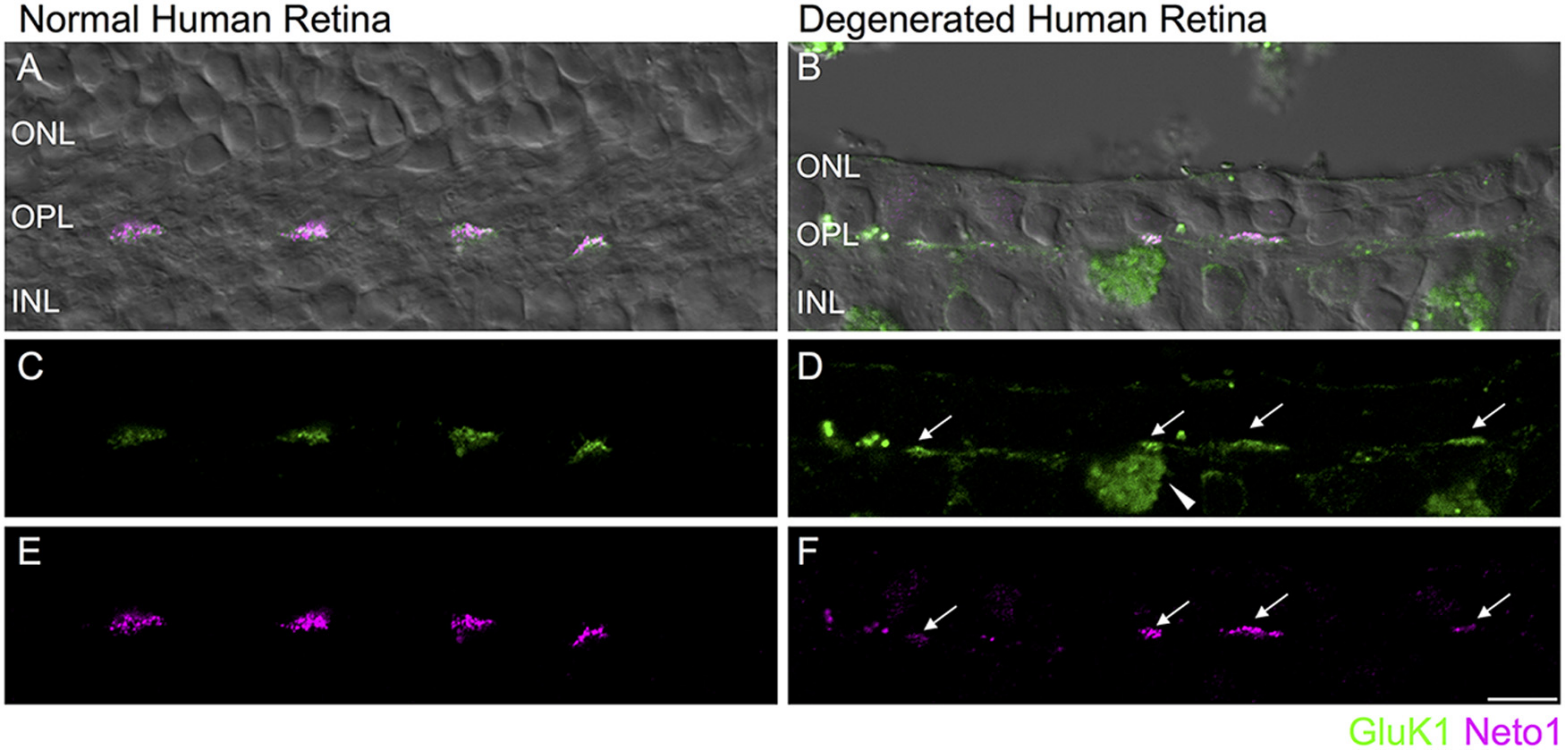
**Retention of kainate receptors after photoreceptor degeneration in human retina.** **(A,B)** Immunolocalization of GluK1 (green) and Neto1 (magenta) in normal human peripheral retina **(A)** and in degenerated human retina **(B)**. The fluorescence image is superimposed on the transmitted light image (gray) to show retinal morphology. **(C,D)** Clusters of GluK1 staining in the normal and degenerated retina (arrows). An autofluorescent pigment epithelial cell is indicated by the arrowhead. **(E,F)** Same region as in **(C,D)** showing clusters of Neto1 staining. Scale bar = 10 μm.

Slides were imaged on an Olympus Fluoview 1000 confocal microscope with a 60x/1.42 N.A. oil immersion objective. The red and green color channels were acquired with sequential scanning to obviate any possibility of cross-talk between fluorophores. Transmitted light images were collected together with the fluorescence images to show retinal morphology. Images were pseudocolored and adjusted for brightness and contrast using Image J and Adobe Photoshop CC 2014. Confocal images are z-projections of three (**Figure 6**) to five (**Figures 1–5**) confocal sections spanning a total z-depth of 1.8–2.4 μm (sampled at Nyquist spacing). For fluorescence intensity analysis, confocal stacks were acquired from *wild-type* and *rd10* retinae. Optimal acquisition parameters were determined so that intensity values were within the linear range for both channels and both genotypes – acquisition parameters were then kept constant for all images. For image analysis, confocal z-planes were projected with maximum intensity and polygonal regions-of-interest (ROI) were drawn around clusters of GluK1/Neto1 staining. The mean intensity was determined by finding the average intensity per unit area of the ROIs after subtraction of mean background fluorescence. Per this metric, a mean intensity increase could result either from an increase in intensity or area of the puncta, or a reduction in the area between puncta. All values are reported as mean ± standard error of the mean (SEM) and statistical comparisons were made using unpaired, two-tailed *t*-tests.

## Results

We used the *rd10* mouse model of retinitis pigmentosa to study the effect of photoreceptor degeneration on bipolar cell morphology and glutamate signaling. *Rd10* mice harbor a missense mutation in the *Pde6b* gene resulting in a rod-cone degeneration that progresses from central to peripheral retina (Chang et al., 2007). We studied retinae from two age cohorts, one at 3–4 months of age (referred to herein as the “early” cohort), when rods have degenerated but some cones remain in the peripheral retina, and an older cohort aged between 8 and 18 months when almost all photoreceptors have degenerated (referred to herein as the “late” cohort). To assess retinas for residual, structurally compromised photoreceptors, we performed immunostaining with recoverin and vGluT1 (see **Figure 3** for recoverin staining). At both timepoints, residual photoreceptors were restricted to peripheral retinal regions. Since we were interested in the impact of de-afferentation on glutamate receptor localization, we focused our investigations on the central retina where few, if any, photoreceptors were expected to be present.

Our first aim was to evaluate the dendritic morphology of cone bipolar cells after extensive rod and cone degeneration. Previous studies of the *rd10* mouse show that there may be time-dependent regression of Off bipolar cell dendrites. At postnatal day (PND) 40, Barhoum et al. (2008) reported that dendrites of recoverin-positive Off bipolar cells (Type 2 bipolar cells) were in contact with residual cones. However, other studies have reported that by 6–9 months of age, the dendrites of these cells were not visible (Gargini et al., 2007; Biswas et al., 2014). Similarly, extensive loss of Type 2 Off bipolar dendrites has also been demonstrated with an alternate marker, znp1 (also known as synaptotagmin 2), in *rd10* animals at 1 year of age (Barone et al., 2014). The dendrites of Gγ13-labeled rod and On cone bipolar cells were largely absent from the outer plexiform layer at post-natal month 3.5 (Phillips et al., 2010). The lack of bipolar cell dendrites observed in previous studies might reflect the loss of marker proteins rather than the loss of cone bipolar dendrites. Moreover, it is unclear whether dendrites are lost from Off bipolar cell types other than the Type 2. Thus, we evaluated dendritic morphology with an alternative cone bipolar cell marker, secretagogin, which labels the dendrites, soma and axon terminals of most Off (Type 2, 3, and 4) and On cone bipolar cells (Type 5, 6, and possibly 8) in mouse retina (Puthussery et al., 2010). In wild-type mice, the dendrites of secretagogin-labeled cone bipolar cells terminated in regular clusters, which likely correspond to the location of cone pedicles (see **Figures 1A,G,J**). In *rd10* mice, secretagogin-labeled cone bipolar cell dendrites were evident at both the early and late time-points (**Figures 1B,C,H,I**). The dendrites in *rd10* retina differed from wild-type, forming larger and more disorganized clusters. To determine whether the observed dendrites originated from On or Off cone bipolar cells, we next performed double labeling for secretagogin and Islet-1, the latter of which labels the somata of rod bipolar and On cone bipolar cells (Elshatory et al., 2007). In both wild-type and *rd10* retina, dendrites extended from the somata of Islet-1-negative Off bipolar cells, as well as from Islet-1 positive On cone bipolar cells (**Figures 1D–L**). Together, these results indicate that at least a subset of On and Off cone bipolar cells retain some, albeit disorganized, dendritic processes after de-afferentation.

Depending on species, most or all Off bipolar cells receive input through kainate-type glutamate receptors (Buldyrev et al., 2012; Puller et al., 2013; Lindstrom et al., 2014; Puthussery et al., 2014). Thus, our next goal was to assess kainate receptor expression in Off cone bipolar cells after extensive photoreceptor degeneration. Kainate receptors are heteromers comprised of at least one obligatory low affinity subunit (GluK1-3) with the possible inclusion of a high affinity subunit (GluK4-5). GluK1 is the major low affinity subunit in mouse retina (Haverkamp and Wassle, 2000; Puller et al., 2013). In addition, the kainate receptor auxiliary subunits, Neto1 and Neto2, modulate the functional properties of kainate receptors and may also be involved in kainate receptor clustering and localization (Copits and Swanson, 2012; Tomita and Castillo, 2012). Since Neto1 is present in the outer retina (Chow et al., 2004; Lindstrom et al., 2014; Puthussery et al., 2014), we used this as an additional marker for kainate receptor containing synapses. It is important to note that it is not known whether GluK1 and Neto1 are colocalized at the same mouse retinal synapses and we did not quantify the extent of colocalization in this study given the limited resolution of confocal microscopy. Rather, we used these markers to localize areas with persistent kainate receptor expression. In wild-type retina, GluK1, and Neto1 were concentrated in discrete clusters of puncta in the outer plexiform layer (**Figures 2A,D,G**). Double labeling of GluK1 with PNA indicated that these clusters correspond to the locations of cone pedicles (data not shown), consistent with previous reports (Haverkamp et al., 2003). In the *rd10* retina, clusters of GluK1 staining were also observed but the staining intensity was approximately 30% of wild-type values (intensity per unit area ROI, *wt* late: 1520 ± 110 vs *rd10* late 462 ± 62, *P* = 0.0011, **Figures 2E,F,J**). GluK1 immunoreactivity also appeared more diffuse and the puncta were qualitatively smaller in *rd10* compared to wild-type retina (**Figures 2D–F**). These results suggest that GluK1 is downregulated and/or re-distributed in Off bipolar dendrites of the *rd10* retina. In contrast to GluK1, strong Neto1 expression was evident in the *rd10* retina, even in the older cohort of animals, and the staining intensity was comparable to wild-type (intensity per unit area ROI, *wt* late: 1215 ± 204 vs. *rd10* late: 936 ± 104, *P* = 0.2901, **Figures 2G–J**). The area of the clusters of GluK1/Neto1 staining was approximately fourfold larger in the *rd10* retina (*rd10* (late) 19.92 ± 2.05 μm^2^ vs. *wild-type* (late) 5.15 ± 0.38 μm^2^, *P* = 0.0021, *n* = 3 retinas of each genotype). Moreover, the overall number of GluK1/Neto1 containing clusters was lower in *rd10* compared to wild-type (average # of clusters per 100 μm length of OPL; wt 8.91 ± 0.25, *rd10* 2.19 ± 0.16, *P* < 0.0001).

To determine whether the large clusters of GluK1/Neto1 staining were associated with remodeled bipolar cell dendrites in *rd10* retina, we double labeled for Neto1 and secretagogin (**Figures 3A–F**). In wild-type retina, Neto1 puncta were localized to the tips of secretagogin-labeled bipolar cell dendrites. In the *rd10* retina, Neto1 puncta were present in irregularly sized clusters that coincided with the location of remodeled bipolar cell dendrites (**Figures 3D–F**). Finally, we confirmed that Neto1 clusters persisted in the absence of photoreceptors by double-labeling for recoverin, a calcium-binding protein associated with rod and cone photoreceptors (**Figures 3G–I**). At the early time-point, recoverin detects residual structurally altered photoreceptors in peripheral *rd10* retina (**Figure 3H**), whereas no photoreceptors were detectable in the central *rd10* retina (**Figure 3I**). Notably, Neto1 expression was observed in central retinal regions where there were no residual photoreceptors (**Figure 3I**). Similar results were obtained when residual photoreceptor terminals were detected by staining for vGluT1 (data not shown). These results indicate that kainate receptors and their auxiliary proteins remain in remodeled Off bipolar cell dendrites even in the absence of presynaptic photoreceptors.

Next, we assessed the localization of TRPM1, the non-selective cation channel coupled to mGluR6 in On bipolar cells (Morgans et al., 2009; Shen et al., 2009; Koike et al., 2010). In wild-type retina, TRPM1 was localized to puncta in the outer plexiform layer as well as in the somata of On bipolar cells, consistent with previous reports (**Figure 4A**) (Morgans et al., 2009; Koike et al., 2010). In the *rd10* retina, punctate TRPM1 staining was absent, but strong somatic expression remained in both the early and late mouse cohorts (**Figures 4B–D**).

Do comparable changes in glutamate receptor signaling occur in the human retina after photoreceptor loss? To address this question, we evaluated a human retinal sample in which photoreceptors had degenerated due to a serous retinal detachment secondary to a choroidal melanoma (see Materials and Methods). In normal human retina, TRPM1 immunoreactivity was localized to the somata and dendritic tips of On bipolar cells (assessed in retinas from two eyes). In accord with previous reports, we noted large puncta corresponding to the dendritic tips of rod bipolar cells as well as clusters of smaller puncta that were localized to the base of PNA-labeled cone pedicles (**Figures 5A,B,E**) (Morgans et al., 2009; Klooster et al., 2011). In contrast, in areas of human retina in which the outer nuclear layer (ONL) was one to two cell bodies thick, punctate immunostaining was absent from the outer plexiform layer but strong staining remained in the On bipolar cell somata and their proximal dendrites (**Figures 5C,D,F**). Although PNA binding was not detectable in this sample, residual photoreceptors could be detected in the ONL with antibodies for recoverin or vGluT1 (data not shown).

Finally, we assessed whether kainate receptor expression persists in the degenerated human outer retina. As in mouse retina, GluK1 and Neto1 localized to discrete clusters in the normal OPL. These clusters were colocalized with PNA (data not shown) suggesting that they are clustered at the base of cone pedicles, as in macaque retina (Puthussery et al., 2014) (**Figures 6A,C,E**). In the degenerated sample, clusters of GluK1 and Neto1 remained in the outer plexiform layer (**Figures 6B,D,F**). Taken together, these results suggest that comparable changes in glutamate receptor signaling occur in mouse and human retina in response to photoreceptor degeneration.

## Discussion

We have shown that the kainate receptor subunit, GluK1, and the kainate receptor auxiliary protein, Neto1, persist in Off bipolar cell dendrites after extensive photoreceptor degeneration. GluK1 expression was diffusely distributed in bipolar cell dendrites in the *rd10* and the intensity of this signal was reduced compared to wild-type retina. How can we reconcile the apparent down-regulation of GluK1 with the preservation of Off bipolar glutamatergic currents in our earlier functional study (Puthussery et al., 2009)? One possibility is that in *rd10* retina, the number of GluK1-containing channels is comparable to wild-type, but the channels no longer form high density clusters. The possibility that up-regulation of another low affinity kainate receptor subunit (i.e., GluK2 or GluK3) compensates for the loss of GluK1 receptors seems unlikely given that we were unable to detect GluK2/3 staining in *rd10* or wild-type outer retina (data not shown), a finding in accord with previous studies in wild-type retina (Puller et al., 2013). Small and variable AMPA-receptor currents have been detected in normal mouse Off bipolar cells (Puller et al., 2013; but see Borghuis et al., 2014), and it is possible that these might be upregulated in *rd10* retina. In this context, it should be noted that On cone bipolar cells in *rd1* retina transiently (from P15 to P40) express aberrant AMPA/kainate glutamate receptors, suggesting that significant glutamate receptor plasticity can occur during degeneration (Chua et al., 2009). Further functional studies, using AMPA and kainate receptor selective agents (Borghuis et al., 2014; Puthussery et al., 2014), are needed to assess bipolar cell glutamate receptor function during the course of the degenerative process.

Perturbations in On bipolar cell signaling have been described in numerous models of retinal degeneration. In particular, loss of synaptic mGluR6 seems to be a ubiquitous finding (Strettoi and Pignatelli, 2000; Cuenca et al., 2004; Gargini et al., 2007; Barhoum et al., 2008). Although some mGluR6 receptors redistribute to On bipolar cell somata in the *rd10* mouse, activation of these channels does not produce significant currents (Puthussery et al., 2009), suggesting that they are either at a very low density, or they are de-coupled from the downstream TRPM1 channel. Compared with mGluR6, the status of TRPM1 after photoreceptor loss is less clear. We have shown that in regions of *rd10* and human retina in which most photoreceptors had degenerated, synaptic TRPM1 expression is lost from On bipolar cell dendritic tips, but expression persists in the soma and primary dendrites. Our results are consistent with the pattern of TRPM1 reported by (Križaj et al., 2010) in the *rd1* retina. An important question is whether somatic TRPM1 channels are in the open or closed state after de-afferentation. Since mGluR6 activation leads to closure of TRPM1 channels, one might predict that loss of mGluR6 would render the TRPM1 channels constitutively open. However, direct patch-clamp recordings from On bipolar cells in *rd1* mice revealed a relatively hyperpolarized resting potential (∼-50 mV) (Borowska et al., 2011), suggesting that they are in the closed state. Similarly, On bipolar cells in mGluR6-null mice have hyperpolarized resting potentials (Xu et al., 2012), suggesting that extrasynaptic TRPM1 channels are closed. The continued presence of somatic TRPM1 channels might be exploited as part of a treatment strategy if a mechanism could be introduced to enable light-evoked gating of these cation channels.

What are the mechanisms that lead to bipolar cell dendritic remodeling and glutamate receptor down-regulation? This issue has been challenging to resolve given the slow and progressive nature of inherited degenerations. However, a recent study showed that laser-mediated photoreceptor ablation leads to loss of dendritic mGluR6 receptors within hours of injury (Dunn, 2015). These changes precede overt dendritic remodeling and are not prevented by pharmacological activation or blockade of glutamate receptors, suggesting that structural integrity of photoreceptors may be required for synaptic maintenance. Alternatively, it is possible that more physiological patterns of glutamate stimulation are necessary to maintain synaptic mGluR6 expression (Pottackal, 2015). Further studies are needed to determine whether reductions in kainate receptor expression occur on a similar timescale to that of mGluR6 receptors.

Could synaptic glutamate receptor expression be restored upon photoreceptor repair or replacement? In support of this idea, Sher et al. (2013) demonstrated restoration of retinal structure and function in regions where patches of photoreceptors had been laser ablated. In this case, inputs to de-afferented bipolar cells were restored when photoreceptors from outside the ablation zone migrated into the lesioned region. Moreover, as mentioned above, the aberrant ionotropic glutamate receptor expression observed in On bipolar cells of the *rd1* retina implies that significant glutamate receptor plasticity is possible. A recent study in a mouse model of X-linked retinoschisis showed that restoration of glutamate receptor function can also occur in On bipolar cells (Ou et al., 2015). In this model, synaptic localization of TRPM1 was reinstated after AAV-mediated gene therapy to restore retinoschisin 1 expression, indicating a capacity for synaptic plasticity after disassembly. However, it is important to note that mGluR6 is preserved in the retinoschisin model, and photoreceptors do not degenerate. Thus, the findings from this model may not necessarily apply to all models of inherited retinal degeneration. Overall, the available evidence suggests that the retina retains some capacity for synaptic repair and rewiring after de-afferentation.

Bipolar cells are attractive targets for vision restoration, since they are preserved in late stages of photoreceptor degeneration and offer the potential to restore greater spatiotemporal processing than ganglion cell or non-targeted therapies (Gaub et al., 2014). For example, light-activated microbial opsins and light-gated photoswitches have been targeted to On bipolar cells using the *Grm6* promoter, which drives mGluR6 expression (Lagali et al., 2008; Doroudchi et al., 2011; Cronin et al., 2014; Gaub et al., 2014). A promising direction to extend this approach would be to target an inhibitory channel such as halorhodopsin to Off bipolar cells, thereby reinstating Off and On pathway signaling. However, at present there are no selective promoters for Off bipolar cells. GluK1 and Neto1 show restricted expression in macaque (Haverkamp et al., 2001; Puthussery et al., 2014). and human Off bipolar cells. Thus, promoters for the genes encoding these proteins might be useful for restricting treatments to the Off bipolar cell population.

## Author Contributions

TP and JG designed study and collected data, TP wrote the manuscript.

## Conflict of Interest Statement

The authors declare that the research was conducted in the absence of any commercial or financial relationships that could be construed as a potential conflict of interest.
